# Effects of different maturity status on change of direction performance of youth tennis players

**DOI:** 10.5114/biolsport.2023.121324

**Published:** 2022-12-13

**Authors:** Jaime Fernandez-Fernandez, Jose Canós-Portalés, Rafael Martínez-Gallego, Francisco Corbi, Ernest Baiget

**Affiliations:** 1 Department of Physical Activity and Sports Sciences, Universidad de León, Spain; 2 AMRED, Human Movement and Sports Performance Analysis, Universidad de León, Spain; 3 National Institute of Physical Education of Catalonia, University of Barcelona, Barcelona, Spain; 4 Faculty of Physical Activity and Sport Sciences, University of Valencia, Valencia, Spain; 5 National Institute of Physical Education of Catalonia, University of Lleida, Lleida, Spain

**Keywords:** Peak height velocity, Racket sport, Static start, Rolling start, Change of direction deficit

## Abstract

The aim of this study was to examine the maturational status (i.e., peak height velocity [PHV]) differences in neuromuscular performance (i.e., vertical jump, linear sprint, change of direction (COD) using different tests, and change of direction deficit [CODD]) of young tennis players. One hundred and two tennis players (70 boys and 52 girls; age 13.9 ± 2.0 years, body mass 53.3 ± 12.7 kg, height 163.1 ± 11.9 cm) participated in the study and were divided into Pre-PHV (n = 26), Circa-PHV (n = 33) and Post-PHV (n = 43) groups. Testing included speed (5, 10, and 20 m), COD tests (i.e., modified 5-0-5, pro-agility and hexagon), and bilateral/unilateral countermovement jump (CMJ). Pre- and Circa-PHV players presented lower levels of performance in jumping ability (i.e., both bilateral and unilateral CMJs; *P* < 0.001; ES: 0.85 to 0.98), linear sprints (5 to 20 m; *P* < 0.05 to < 0.001; ES: 0.67 to 1.19) and COD ability tests (modified 5-0-5 test, pro-agility and hexagon) compared to the Post-PHV players. Moreover, Pre-PHV players presented lower CODD% (*p* < 0.05; ES: 0.68–0.72) than Post-PHV for both forehand and backhand sides, and Circa-PHV showed lower values in the CODD of the rolling situation to the forehand side (*p* < 0.05; ES: 0.58). Among the COD tests, the pro-agility test seems to be a simple, easy-to-implement and reliable test, which can provide interesting information about the COD with higher entry speeds. Moreover, specific training strategies related to the PHV and focused not only on the neuromuscular training and COD workouts, but also on maximizing motor skill proficiency, should be recommended.

## INTRODUCTION

The design and implementation of athletes’ training programmes should be related to the physical requirements as well as movement demands of the sport [1]. In this regard, tennis-specific research has explored the activity profile and physiological demands of tennis movement extensively [2–4], highlighting tennis as an intermittent sport that involves short bursts of intense activity (i.e., accelerations, decelerations, changes of direction (CODs), and strokes), during a variable period of time (i.e., to the best of 3 sets in junior levels, up to 5 sets in the Grand Slam tournaments) [5]. Previous research has shown that 80% of all tennis strokes are played covering less than 2.5 m, and fewer than 5% of strokes require more than 4.5 m between strokes [6], suggesting that accelerations, decelerations and CODs are of primary importance in tennis in comparison with maximum sprinting speed [7]. CODs, including deceleration followed immediately by reacceleration of the entire body or an individual body segment [4], occur at nearly every point in tennis and can be considered one of the most important physical skills needed to be a successful tennis player at any level.

Although every tennis point is different, and COD performance depends on a complex interaction of contributing movement variables such as acceleration, entry velocity, distance covered, and degree of change [8], its relevance is well established [9, 10]. However, perhaps due to this complexity, research into the tennis-specific COD is limited, with the earliest research describing players performing 1 to 4 four CODs per point [11]. More recently, and with the use of player tracking technology (i.e., Hawk-Eye), research reported an average of 5 CODs per point, with some variation depending on the participant’s level and sex [4]. Thus, in a more complex analysis [12, 13], it was concluded that professional male and female tennis players performed around 2 CODs during a typical point, characterized by medium to high intensity, and involving basically lateral COD, supporting previous observations [6, 14], highlighting the lateral nature of the tennis player’s movement. As a result, in a competitive match, it is not uncommon for players to perform between 250 and 400 CODs [12].

The evolution of technical and tactical performance in tennis has been accompanied by a progressive increase in game speed [15], which in turn forces athletes to play closer to the margins of the court from an early age [4]. Thus, COD performance has been considered one of the most important physical qualities in the sport, with a recent study showing that COD speed was strongly related to tennis performance (i.e., ranking position) in a sample of 1434 youth tennis players (i.e., 11–17 years). However, tennis-specific information about COD testing and training is still scarce, with very few studies analysing this physical quality [16–18], as well as differences among levels, ages and sexes [19].

Testing the COD ability has received much attention in the last few years, with a wide variety of tests that measure COD ability [20] and employed in different intermittent sports, including tennis [10]. In general, protocols differ in terms of complexity and duration, and the selection of a COD test will depend on the athlete, sport and stage of development [21]. The ‘505 change of direction test’, which requires players to sprint 5 m, turn 180°, and sprint a further 5 m, including a 5 m flying start [22], is probably the most popular test used in intermittent sports. Based on this test, and its comparison with linear sprints (e.g., 5 to 20 m), Nimphius et al. [23] introduced the concept of COD deficit (COD_def_), representing the additional time or velocity that a COD requires when compared with a linear sprint over an equivalent distance (e.g. 10-m time vs. 505 time). More recently, a new standardization of the COD_def_ calculation was presented, based on the percentage difference between COD and linear sprint tests (i.e., CODD%) [24]. Researchers suggested that the use of CODD% may be advantageous for practitioners as it reports CODD in a comprehensive, uniform, and consistent manner.

Depending on the sport-specific needs, COD tests may require a low-velocity COD, such as the modified 505 test (i.e., using a stationary start); a high-velocity COD, such as the traditional 505; or a test such as the pro-agility test, which requires both a low-speed and high-speed COD that can be split [25]. In this regard, since CODs occur from various distance approaches in sport, it seems interesting to assess not only the COD_def_ following a static start (i.e., using the modified 505 test), but also the COD_def_ following a rolling start, characterized with higher entry speeds [25]. To the best of our knowledge, only one previous study has used the pro-agility test as a measurement tool in tennis players, reporting that the test was a reliable and valid test for use in competitive junior tennis players [26]. However, there is no information about the relationship between this test and linear sprint, other COD tests and jump performances in tennis players across different maturational statuses. By providing normative data, it enables practitioners to identify, monitor and develop players’ performance through effective strategies for improving COD ability.

Therefore, the aim of this study was to examine the maturational status (i.e., peak height velocity [PHV]) differences in neuromuscular performance (i.e., vertical jump, linear sprint, COD using different tests, and change of direction deficit [CODD]) of young tennis players.

## MATERIALS AND METHODS

### Design

The current investigation is an observational and descriptive analysis to determine maturational status differences in measures of linear sprint, jumping performance, and COD ability in youth tennis players. Testing protocols were conducted over a 4-week period beginning at the end of February 2022, and sessions were undertaken between 12:00 pm and 5:00 pm, with the players being tested at their respective tennis clubs. All tests were performed in the same order using the same testing devices, measurement protocols, and experienced evaluators. The testing took place on an outdoor synthetic court (temperature, 17.5–24 °C; relative humidity, 60–66.0%; Kestrel 4000 Pocket Weather Tracker, Nielsen Kellerman, Boothwyn, PA). Subjects were instructed to avoid all sources of caffeine for 24 h before testing and to have their habitual breakfast at least 3 h before the start of measurements.

### Subjects

One-hundred and twenty-two junior tennis players (70 boys and 52 girls; age 13.9 ± 2.0 years, body mass 53.3 ± 12.7 kg, height 163.1 ± 11.9 cm, estimated age at peak height velocity (PHV) 13.5 ± 1.1 years) took part in this study (Table 1). The sample was composed of competitive players with similar competitive levels and technical abilities from eight different tennis clubs selected by the coaching staff. All players followed similar training schedules, including mixed participation (boys and girls training at the same time), and the organization of training groups based on the chronological ages (U13 and U15). The staff of the different clubs sent an electronic document with information about the number of injuries and the number of training hours. Thus, players included in the study completed 10.3 ± 4.4 h of combined tennis and physical training per week and had a minimum training background of 4.2 years. None of the players reported any history of chronic medical conditions during the previous 12 months. Before taking part in the study, the subjects and their parents/guardians were fully informed about the study protocol and provided their written informed consent. The Spanish Tennis Federation Ethics committee approved the procedures (RFET.EC_21.3) in accordance with the latest version of the Declaration of Helsinki.

**TABLE 1:** Descriptive characteristics of junior tennis players according to their maturational groups.

	Players (n = 102)	Pre-PHV (n = 26)	Maturational groups Circa-PHV (n = 33)	Post-PHV (n = 43)
Chronological age (years)	13.9 ± 2.0	11.9 ± 1.2^$^^###^	13.3 ± 1.1^###^	15.6 ± 1.6
Height (cm)	163.1 ± 11.9	150.8 ± 7.2^$$^^###^	162.1 ± 8.2^###^	171.3 ± 9.4
Body mass (kg)	53.3 ± 12.7	41.6 ± 7.9^$^^###^	51.1 ± 9.2^##^	62.1 ± 11.0
APHV (years)	13.5 ± 1.1	13.8 ± 0.9	13.4 ± 1.2	13.4 ± 1.1
Maturity offset (years)^&^	0.4 ± 1.8	-1.9 ± 0.7^$$^^###^	-0.1 ± 0.6^###^	2.2 ± 1.0
Training volume (h · week^-1^)	10.3 ± 4.3	8.9 ± 4.2^#^	9.7 ± 4.1	11.4 ± 4.4

The values presented are means ± SD. Pre-PHV: pre peak height velocity group; Circa-PHV: around peak height velocity group; Post-PHV: post peak height velocity group; APHV: Estimated age at peak height velocity.

& Estimation of years from predicted PHV.

$ significantly different from Circa-PHV group (*p* < 0.05).

$$ significantly different from Circa-PHV group (*p* < 0.001).

# significantly different from Post-PHV group (*p* < 0.05).

## significantly different from Post-PHV group (*p* < 0.01).

### significantly different from Post-PHV group (*p* < 0.001).

### Procedures

#### Maturity status

Body height was measured using a fixed stadiometer (± 0.1 cm; Holtain Ltd., Crosswell, UK), sitting height with a purpose-built table (± 0.1 cm; Holtain Ltd., Crosswell, UK), and body mass with a digital balance (± 0.1 kg; ADE Electronic Column Scales, Hamburg, Germany). Pubertal timing was estimated according to the biological maturation of each individual using a predictive equation previously described in the literature [27]. The age of peak linear growth (age at peak height velocity [APHV]) is an indicator of somatic maturity representing the time of maximum growth in stature during adolescence. Maturity offset (MO) was achieved in a non-invasive manner using the regression equation previously proposed [28]. Moreover, to account for the reported error, players were grouped into discrete bands based on their MO (pre-PHV [< -1], Circa-PHV [-0.5 to 0.5], post-PHV [> 1]), and players with a maturity offset from -1 to -0.5 (n = 10) and 0.5 to 1 (n = 20) were removed from the dataset when players were analysed by stage of maturation. Overall, 102 players (63 boys and 39 girls) were finally included in the study.

#### Countermovement jump (CMJ) test

A bilateral CMJ and unilateral (i.e. dominant [CMJD] and non-dominant [CMJND] side) CMJs were performed using an OptoJump photoelectric system (Microgate, Bolzano, Italy) and according to the protocol previously described [10]. During the jump, hands were held at the hips to minimize the influence of the upper body on jump performance. From a standing position with straight knees, players squatted down to ~90º and accelerated at maximal velocity in a vertical direction. Each player performed 3 maximal attempts interspersed with 45 s of passive recovery, and the highest jump was recorded and used for statistical analysis.

#### Sprint test

Time during a 20-m linear sprint (with 5 and 10 m split times) was measured by means of single beam photocell gates placed 1.0 m above the ground level (Witty System, Microgate, Bolzano, Italy). Each sprint was initiated 0.5 m behind the first photocell gate, which then started a digital timer. Players started the linear sprint test in a standing split position, with their preferred foot behind the starting line, followed by accelerating forward at maximal effort until they passed the last photocell gate placed at 20 m. Each player performed three maximal 20-m sprints with at least two min of passive recovery in between the trials, and the average performance was calculated.

#### Modified 505 COD test

**FIG. 1:**
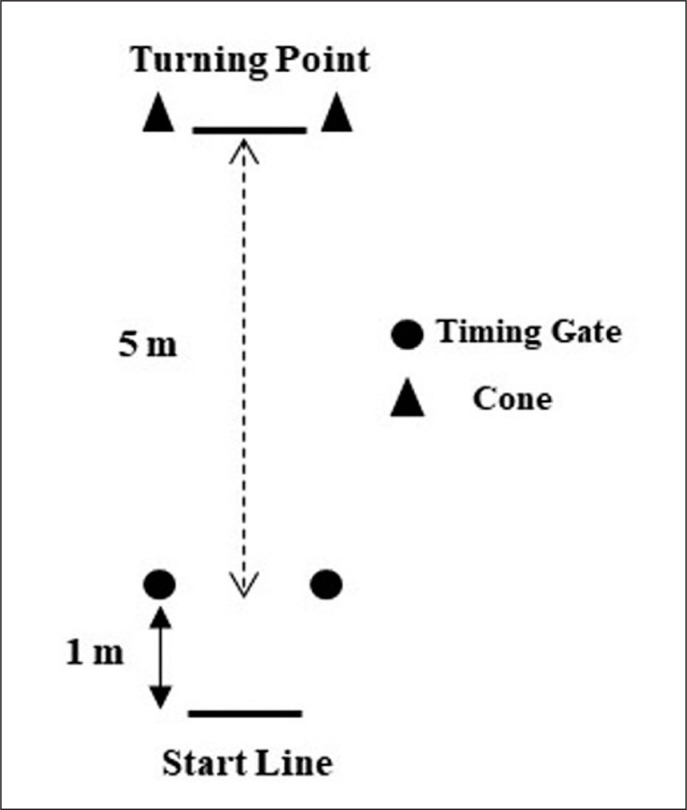
Structure and dimensions of the modified 505 change of direction test. Note: m = meters.

The abilities of the athletes to perform a single, rapid 180° change of direction over a 5 m distance was measured using a modified version (stationary start) of the 505 COD test [10] (Figure 1). Players started in a standing position with their preferred foot 0.5 m behind the starting line. They were asked to plant their preferred (i.e., considered as the dominant side) foot on executing the turn. Three trials were completed, and the best time was recorded (Witty System, Microgate, Bolzano, Italy). Two minutes of rest was allowed between trials [23]. The CODD% was calculated following the formula: CODD% t = ([*modified 505 time* – *10-m sprint time*]/*10-m sprint time*)*100 [24].

#### Pro-Agility test

**FIG. 2:**
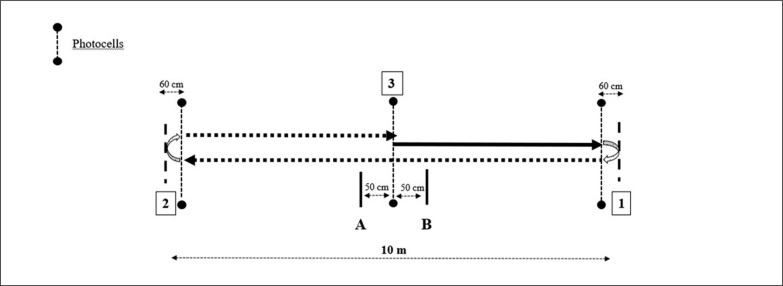
Schematic representation of the pro-agility test. Note: A: Starting line for a right-handed player moving to the forehand side; B: Starting line for a right-handed player moving to the backhand side; 1: Change of direction in the static situation; 2: Change of direction in the rolling situation; 3: Finish

For the pro-agility test, players started from a line placed 0.5 m from the start line, facing perpendicular to the running direction. The test was conducted in the baseline of a tennis court, in order to simulate as much as possible a specific movement. The players were instructed to initiate the trials when they were ready (i.e., self-selected start), sprinted 5 m to the forehand side (i.e., right side for a right-handed player, and considered as the CODD% of the static situation), then 10 m to the backhand side (i.e., left side for a right-handed player, and considered as the CODD% of the rolling situation), and 5 m back to finish the test as they crossed the centreline (Figure 2) [25]. Three trials, interspersed with two min of passive recovery, were recorded for both the forehand and the backhand side, the order of which was randomized. The CODs within each test were conducted on alternative legs (i.e., for a right-handed player, when they ran to the forehand side, players used the right leg on the COD, and the left leg when they ran to the backhand) [9]. A researcher was positioned at each turning line, situated 60 cm after each photocell, and if the player changed direction before hitting the turning point, or performed the COD with the wrong foot, the trial was disregarded and repeated after a recovery period. Both the forehand and backhand side trials were recorded and used for further analysis [29]. Thus, the CODD% was calculated in both static and rolling situations, following the formula: *staticCODD% t* = ([*first 10 m of the pro-agility – 10-m time*]/*10-m sprint time*)*100; and *rolling-CODD% t* = ([*first 10 m of the pro-agility – 10 to 20 m split time*]/ *10 to 20 m split time*)*100.

#### Hexagon test

**FIG. 3:**
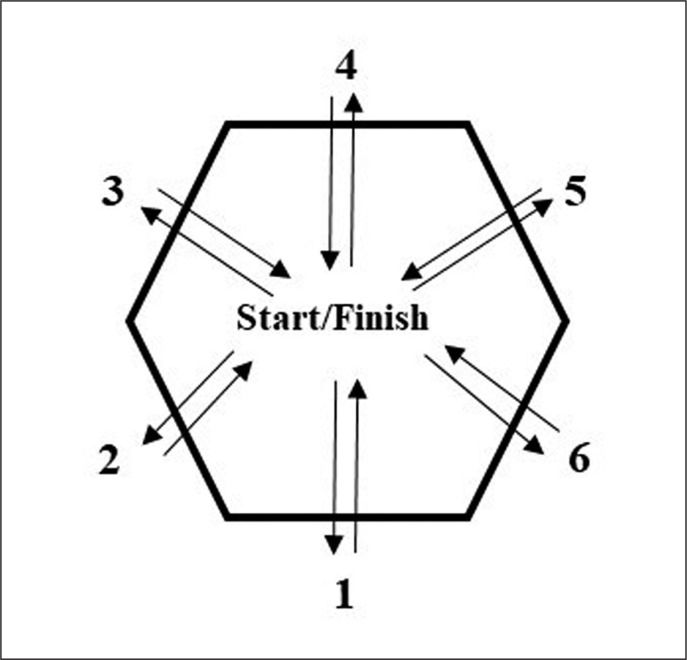
Schematic representation of the hexagon test.

The hexagon test requires the player to stand facing forward in the middle of a hexagon measuring 60 cm per side and with 120-degree angles. With feet together and hips facing forward throughout the test sequence, subjects hopped forwards and backwards in a clockwise manner over each of the six sides of the hexagon, completing three sequences [30] (Figure 3). Each repetition was recorded using a mobile phone (iPhone XS; Apple Inc., Cupertino, CA, USA) running iOS 13.7 that was secured to a small tripod with a mount (GripTight Mount Pro, Joby, USA) and positioned 2 m from the hexagon. All trials were recorded at 240 Hz, and the time to complete three sequences was later analysed with video analysis software (Kinovea version 0.8.15, available for download at: http://www.kinovea.org). A penalty of 0.5 s was given each time the player touched a line, and a 1.0 s penalty was given if the player failed to follow the correct sequence [30]. A practice attempt was given prior to the three attempts used for analyses, with a two min rest between them. The fastest time of three attempts was used for analysis.

#### Statistical analysis

Descriptive statistics (mean and standard deviation) were calculated for each of the variables. The Kolmogorov–Smirnov test was applied to determine whether data sets were normally distributed. Within-session reliability of test measures were assessed using intraclass correlation coefficients (ICC) and the coefficient of variation (CV) with their corresponding 95% confidence intervals, respectively. We considered an ICC < 0.50 as poor, 0.50 ≤ ICC < 0.75 as moderate, 0.75 ≤ ICC < 0.90 as good, and ICC > 0.90 as excellent [31]. Absolute reliability was calculated using the standard error of measurements (SEM), which was calculated as SD × √1 - ICC, where SD is the SD of all scores from the subjects [32]. The SEM was used to calculate the minimal detectable change (MDC) and was calculated as SEM × 1.96 × √2 to construct a 95% CI [32]. A paired t-test was used to assess the potential differences between sides during the pro-agility test, as well as between CODD% in the static and rolling situations. In order to investigate the differences caused by maturation, one-way independent-measures analyses of variance (ANOVAs) were performed, and the Bonferroni post-hoc test was used to aid in the interpretation of the results. Differences between genders were compared using an independent sample *t*-test. Effect sizes (ES) were calculated to estimate the magnitude of differences in the tested variables and interpreted using the following thresholds: < 0.2, trivial; ≥ 0.2 to 0.49, small; ≥ 0.5 to 0.79, moderate; and ≥ 0.8, large [33]. Precise p-values were reported, and the significance level was set at p (probability of type I error) < α = 0.05. All statistical analyses were performed using IBM SPSS Statistics 26.0 (SPSS, Inc., Chicago, IL.).

## RESULTS

The reliability data of the tests conducted in the present study are shown in Table 2. All tests showed acceptable between-trial reliability scores, with CV values between 1.1% and 7.9% and good to excellent ICC (0.824 to 0.990) (Table 2).

**TABLE 2:** Within-session reliability of test measurements.

	ICC (95% CI)	CV (%) (95% CI)	SEM	MDC
***Vertical jumping ability***				
**CMJ (cm**)	0.985 (0.974–0.992)	3.45 (2.65–4.24)	0.76	2.11
**CMJD (cm)**	0.927 (0.843–0.963)	7.86 (6.08–9.65)	1.02	2.82
**CMJND (cm)**	0.962 (0.927–0.980)	6.84 (5.15–8.52)	0.76	2.11

***Linear sprinting ability***				
**5 m (s)**	0.975 (0.948–0.988)	2.34 (1.70–2.98)	0.02	0.04
**10 m (s)**	0.990 (0.981–0.995)	1.36 (0.88–1.95)	0.02	0.04
**20 m (s)**	0.990 (0.982–0.995)	1.09 (0.75–1.42)	0.03	0.09

***Change-of-direction ability***				
**M505 (s**)	0.925 (0.837–0.961)	2.23 (1.77–2.70)	0.10	0.28
**CODD%**	0.957 (0.871–0.986)	2.44 (1.62–3.25)	0.03	0.08
**Hexagon (s)**	0.824 (0.729–0.908)	4.34 (3.43–5.26)	0.35	0.97
**Pro-Agility (s)**	0.849 (0.775–0.898)	2.10 (1.58–2.63)	0.17	0.46
**CODD%_S**	0.874 (0.648–0.960)	3.65 (2.71–4.59)	0.05	0.15
**CODD%_R**	0.940 (0.822–0.980)	2.89 (2.12–3.65)	0.06	0.17

ICC = intraclass correlation coefficient; CV = coefficient of variation; CI = confident interval; SEM = standard error of measurement; MDC = minimal detectable change; D: dominant side; ND: non-dominant side; CMJ: countermovement jump; M505: modified change of direction test; CODD%: Percentage-based COD deficit; CODD%_S: percentage based CODD in the static situation; CODD%_R: percentage based CODD in the rolling situation.

As shown in Table 1, the results showed that Pre-PHV players presented lower training volume values compared to Post-PHV (*p* < 0.05; ES: 0.58), with no differences compared to Circa-PHV players. In addition, Pre-PHV players also showed smaller body height (*p* < 0.001; ES: 1.5 and 2.4) and body mass (*p* < 0.001; ES: 1.1 and 2.1) values together with smaller MO (*p* < 0.001; ES: 2.8 and 4.7) compared to Circa-PHV and Post-PHV players, with no differences regarding APHV.

Table 3 shows the comparisons of the performance tests according to the maturational status. The between-group analysis revealed significant differences (*p* < 0.001 to 0.003) in all tests performed (i.e., jumps, linear sprinting, and COD). Post-hoc analyses revealed that compared to Post-PHV, Pre-PHV players were significantly slower (*p* < 0.001; ES: 0.76 to 1.19) in linear sprints (5 to 20 m) and all COD tests (M505, pro-agility and hexagon). Moreover, they showed significantly lower (*p* < 0.001; ES: 0.85 to 0.98) performance levels in all jumps (CMJ, CMJD, and CMJND). In addition, compared to Circa-PHV, Pre-PHV players also demonstrated moderately lower performances in the hexagon test (*p* < 0.05; ES: 0.6). Regarding the CODD% and CODD% in the static situation, no differences were found between groups. However, data showed significant differences in the CODD% of the rolling situation, with Pre-PHV players presenting lower CODD% (*p* < 0.05; ES: 0.68–0.72) than Post-PHV for both forehand and backhand sides.

**TABLE 3:** Differences between maturational groups on vertical jump, linear sprint and change of direction abilities.

	Maturational groups			One-way ANOVA		Effect size (90% IC)	

	Pre-PHV (n = 26)	Circa-PHV (n = 33)	Post-PHV (n = 43)	*P*-value	Pre-PHV vs. Circa-PHV	Circa PHV vs. Post-PHV	Pre-PHV vs. Post-PHV
***Vertical jumping ability***							

**CMJ (cm)**	25.0 ± 4.3^$$^	24.6 ± 4.8^$$$^	30.4 ± 6.5	< 0.001	-0.09 (-0.53–0.34)	0.98 (0.58–1.38)	0.98 (0.54–1.41)

**CMJ D (cm)**	12.3 ± 3.2^$$^	13.0 ± 2.9^$$$^	16.4 ± 3.9	< 0.001	-0.07 (-0.51–0.36)	0.98 (0.58–1.38)	0.89 (0.46–1.31)

**CMJ ND (cm)**	13.5 ± 3.2^$$^	13.1 ± 3.0^$$$^	16.6 ± 4.1	< 0.001	-0.11 (-0.54–0.33)	0.96 (0.56–1.36)	0.85 (0.42–1.27)
***Linear sprinting ability***							

**SP5 (s)**	1.25 ± 0.1^$$^	1.23 ± 0.1^$$^	1.12 ± 0.2	< 0.01	-0.21 (-0.65–0.33)	-0.73 (-1.12– -0.34)	-0.85 (-1.27–0.42)

**SP10 (s)**	2.15 ± 0.14^$$^	2.09 ± 0.13^$$^	1.90 ± 0.33	< 0.001	-0.35 (-0.78–0.09)	-0.76 (-1.15– -0.37)	-0.93 (-1.36– -0.50)

**SP20 (s)**	3.79 ± 0.27^$$$^	3.67 ± 0.24^$$^	3.29 ± 0.60	< 0.001	-0.48 (-0.92– -0.04)	-0.84 (-1.23– -0.44)	-1.08 (-1.52– -0.64)
***Change-of-direction ability***							

**M505 (s)**	3.07 ± 0.22^$^	3.03 ± 0.17^$^	2.78 ± 0.49	0.003	-0.21 (-0.65–0.23)	-0.68 (-1.06– -0.29)	-0.76 (-1.18– -0.33)

**CODD%**	42.94 ± 5.90	44.5 ± 6.1	45.52 ± 8.72	0.422	0.26 (-0.20–0.72)	0.24 (-0.21–0.68)	0.44 (0.00–0.89)

**ProAgilityF (s)**	6.69 ± 0.47^$$$^	6.59 ± 0.36^$$$^	6.21 ± 0.33	< 0.001	0.24 (-0.68–0.20)	-1.09 (-1.49– -0.68)	-1.10 (-1.62– -0.74)

**CODD%_F_S_**	60.5 ± 6.6	62.6 ± 7.9	64.0 ± 9.9	0.326	0.28 (-0.18–0.75)	0.16 (-0.29–0.60)	0.41 (-0.03–0.86)

**CODD%_F_R_**	100.3 ± 12.7*^$^	102.4 ± 11.9*^$^	113.7 ± 24.5*	0.011	0.17 (-0.29–0.63)	0.58 (0.13–1.03)	0.68 (0.23–1.14)

**ProAgilityB (s)**	6.62 ± 0.45^$$$^	6.62 ± 0.39^$$$^	6.18 ± 0.34	< 0.001	-0.00 (-0.43–0.44)	-1.20 (-1.61– -0.79)	-1.10 (-1.54– -0.67)

**CODD%_B_S_**	61.3 ± 12.2	63.6 ± 8.1	64.2 ± 10.8	0.569	0.22 (-0.29–0.63)	0.07 (0.13–1.03)	0.25 (0.23–1.14)

**CODD%_B_R_**	95.6 ± 25.9*^$^	101.7 ± 22.5*	111.6 ± 18.1*	0.020	0.25 (-0.21–0.72)	0.49 (0.04–0.93)	0.72 (0.27–1.17)

**Hexagon (s)**	10.74 ± 1.17^†$$$^	10.18 ± 0.61^$^	9.70 ± 0.50	< 0.001	-0.60 (-1.05– -0.16)	-0.87 (-1.26– -0.48)	-1.16 (-1.60– -0.72)

he values presented are means ± SD. F- and P-values were obtained by One-Way ANOVA (3 maturational groups). Pre-PHV: pre peak height velocity group; Circa-PHV: around peak height velocity group; Post-PHV: post peak height velocity group; 90% IC: 90% interval confidence; D: dominant side; ND: nondominant side; CMJ: countermovement jump; SP5: 5-m linear sprint; SP10: 10-m linear sprint; SP20: 20-m linear sprint; M505: modified change of direction test.; CODD%: Percentage-based change of direction (COD) deficit; ProAgilityF: Pro-agility test to the forehand side; ProAgilityB: Pro-agility test to the backhand side; CODD%_F/B_S_: percentage-based COD deficit in the static situation to the forehand/backhand; CODD%_F/B_R_: percentage-based COD deficit in the rolling situation to the forehand/backhand.

† Different from Circa-PHV group (*p* < 0.05).

$ Different from Post-PHV group (*p* < 0.05).

$$ Different from Post-PHV group (*p* < 0.01).

$$$ Different from Post-PHV group (*p* < 0.001).

* Different from CODD% in the static situation (*p* < 0.001).

When comparing Circa-PHV and Post-PHV players, the results also showed significantly lower values in all the variables analysed (Vertical jumping ability: *p* < 0.001; ES: 0.95 to 1.02; Linear sprinting ability: *p* < 0.01; ES: 0.73 to 0.85; COD ability: *p* < 0.05 to < 0.001; ES: 0.67 to 1.11), and CODD% in the rolling situation to the forehand side (*p* < 0.05; ES: 0.58). No differences were found in the CODD%, CODD% in the static situation, for both forehand or backhand sides, and CODD% in the rolling situation for the backhand side.

## DISCUSSION

The aim of this study was to examine the maturational status (i.e., PHV) differences in neuromuscular performance (i.e., vertical jump, linear sprint, COD using different tests, and the percentage-based COD deficit (CODD%) in both static and rolling situations), of young tennis players. The results obtained showed that Pre- and Circa-PHV players presented lower levels of performance in jumping ability (i.e., both bilateral and unilateral CMJs), linear sprints (5 to 20 m), and COD ability tests (modified 5–0–5 test, pro-agility and hexagon) compared to the Post-PHV players. Regarding the CODD% and CODD% in the static situation, no differences were found between groups. However, data showed significant differences in the CODD% of the rolling situation, with Pre-PHV players presenting lower CODD% (*p* < 0.05; ES: 0.68–0.72) than Post-PHV for both forehand and backhand sides, and Circa-PHV showing lower values in the CODD% of the rolling situation to the forehand side (*p* < 0.05; ES: 0.58).

When comparing the maturational status differences in neuro-muscular performance (i.e., vertical jump, linear sprint, COD using different tests, and their associated CODD%) of the tennis players analysed here, the results showed significant differences (ES ranging from 0.76 to 1.19) in all tests performed (i.e., jumps, linear sprinting, and COD), with Pre- and Circa-PHV players showing lower performance levels than their Post-PHV peers. Compared to Circa-PHV, Pre-PHV players showed no differences in any test, except in the hexagon test, with moderately lower performances (ES = 0.60).

As suggested in previous research, in sports in which the training environments are characterized by mixed participation (i.e., boys and girls training together), the use of an athlete’s PHV instead of the chronological age seems to be a better measure to design athletic training programmes [34–36], as it is well known that maturation can influence many aspects of physical performance (i.e., speed, COD, and/or jumping ability) [37]. Pre-PHV players of the present study were significantly smaller (~ 7% to 12%) and presented lower body height (~ 18% to 30%) than Circa- and Post-PHV players, factors that can lead to differences in strength/power and therefore in physical tests. Interestingly, Pre- and Circa-PHV players of this study presented lower levels of jumping (~15–20%) and linear sprinting performance (~ 7–12%), compared to the Post-PHV. Regarding jumping ability, the results were in agreement with previous research conducted with similar tennis populations [19, 35], although the data related to linear sprinting showed no differences between Pre- and Circa-PHV players. This is contrary to previous research, including that on tennis populations [35, 38, 39], suggesting that the time around PHV could be a key point in the improvement in speed in young tennis players, which continues to increase, although not significantly, until Post-PHV stages. The lack of differences between Pre- and Circa-PHV groups here could be related to several factors. First, Circa-PHV subjects are taller and heavier than their Pre-PHV peers, so it seems that the possible benefits of a more advanced maturation are not clear for the Circa-PHV players. In this regard, since around the ages of PHV there is a disproportional growth and disruption of motor coordination in complex motor coordination tasks (i.e., “adolescent awkwardness”) [39], we could speculate that these alterations (i.e., the regulation of the lower extremity joint stiffness [40]) may temporarily compromise the player’s performance levels. Furthermore, when analysing training volumes, there were no differences between groups, which can be related to a bias directed towards a sport-specific activity at the expense of global motor skill training. Thus, the inclusion of training programmes that incorporate a variety of essential motor skills (i.e., locomotion, stabilization) seems to be an essential strategy from which youth athletes can maximize their motor skill proficiency and reduce the risk of sustaining acute and overuse injuries [36].

The regular assessment of physical and technical capacities considered crucial for sporting success is critical for developing tailored and effective training programmes [41]. For this reason, the implementation of practical tests able to provide valid and reliable measures is highly recommended. Results of the COD tests conducted in this study showed acceptable between-trial reliability scores and good to excellent ICC values (CV < 5%; ICC > 0.80). Since the tennis-specific literature related to the pro-agility test is scarce, its implementation in the tennis fitness batteries seems to be interesting. The present results showed that it is a highly reliable measurement, with similar scores to those of one of the most frequently used COD tests (i.e., 505 COD test). Values reported here are also in line with a previous study analysing a very similar test (i.e., the 20-yard shuttle test) in tennis players [18, 26], or studies analysing the pro-agility tests as a measure of COD ability and its utility in sports requiring multiple high degree directional changes, as a measure of repeated 180° COD ability [42, 43]. Furthermore, it should be highlighted that differences between Pre- and Circa-PHV compared to Post-PHV players were significant, with large ES, suggesting that this test is highly sensitive to discriminate between athletes from different maturational stage and a viable tool for talent identification. This hypothesis should be tested in future studies, as previous research has already shown that other specific tennis tests may be able to differentiate between players from different technical levels and ranking positions [18, 44].

In the last few years, and as a practical attempt of removing the confounding factor of large amounts of linear sprinting during the COD tests [20], the CODD has been introduced in the testing batteries of different sports, including tennis [10]. The present results showed that faster players in linear sprints and the M505 test did not present larger COD deficits, with CODD% scores obtained showing that players were 42–45% slower when performing the modified 505 test than when completing an equal distance during a linear sprint. Although this is in line with a recent study conducted with U13 and U15 male and female tennis players [10], the results are contrary to a very recent study conducted with a similar population, including different maturational groups, and showing that Circa-PHV and Post-PHV players who were the faster players in linear sprints presented higher CODD (11–14%) [35]. The differences may be related to the group configuration (i.e., age-grouped instead of biological) or to the CODD analysis (i.e., using percentages or seconds). Although faster tennis players will possibly have greater inertia and thus will need to apply higher breaking forces over longer ground contact times [45], the modified 505 test seems not enough to achieve considerable velocities.

Since tennis movement is mainly characterized by lateral movements, with 60–70% of all COD being > 105º [8], and players braked or decelerated at ≥ 3 ms^-2^ when entering medium and high intensity (i.e., > 2.5 m.s^-1^) direction changes [12], it seems necessary to include high-velocity CODs in the regular testing of these athletes [20]. The inclusion of a rolling start (i.e., an increased entrance velocity), such as the one conducted during the pro-agility test, can help to determine how much time it “costs” an athlete to change direction [29]. The results of the present study showed that first, the CODD in the static situation obtained during the pro-agility test was significantly higher (*p* < 0.001; ES ranging from 2.0 to 3.0) than the CODD during the modified 505 test. To the best of our knowledge, there is no previous research analysing these measures, making comparisons difficult. Although no biomechanical analyses were conducted here, during a 180º COD players orientate their bodies in the direction of travel, while during the pro-agility test, players seem to enter and exit the COD with their bodies orientated down the court (i.e., toward the net) [46]. This can lead to a different entry speed and angle, altering therefore the “cost” of the COD. In this regard, recent research has highlighted several biomechanical differences among tasks with different COD angles [47], such as an increased ground contact time with increased angles during the COD. However, this is speculative, and more research is needed in this regard, including the measurement of ground reaction forces, as well as entry and exit speeds.

Regarding the CODD% in the rolling situation during the pro-agility test, the results showed that, compared with the CODD% in the static situation, an increased entrance velocity, caused by the distance run (i.e., 10 m), significantly exacerbated the CODD (*p* < 0.001; ES ranging from 2.0 to 3.0), meaning that players were more than 100% slower when performing the second part of the pro-agility test than when completing an equal distance during a linear sprint. Although this can be considered an effect of the test procedure (e.g., test position, including static/rolling situations), the results can illustrate the low capacity of these players to effectively decelerate and change direction with an increase in entry velocity [29]. In this regard, since the ability to decelerate efficiently is underpinned by eccentric strength capacity [48], it may indicate that youth athletes might benefit from focusing on deceleration training and increasing the eccentric strength to improve CODD. Moreover, and although the eccentric lower-limb load was not quantified, the accumulation of medium to high velocity COD during the competitive season can be considered a very interesting topic since intense CODs are associated with an increased lower limb load [47]. In this regard, the frequent lower limb joint loading of COD may represent a key risk factor for consideration in the management of hip and knee injuries, which are considered among the most common musculoskeletal complaints in tennis [49]. Future research should analyse the relationship between eccentric strength training and deceleration capabilities, including the CODD.

We recognize that this research had several limitations, include its cross-sectional design and the need to include larger samples with higher performance levels, which could help to avoid potential selection bias. A more detailed analysis of the training volume (i.e., strength, endurance, and/or other qualities) would also help to clarify whether performance differences are also mediated by training. Another limitation was the lack of strength/power related measurements, which would definitely help to determine whether the differences found herein are mediated by differences in the strength levels or in the ability to change direction rapidly. However, we believe that the present design has high levels of ecological validity and may offer a starting point to suggest practical applications to tennis professionals.

## CONCLUSIONS

In conclusion, the present research showed that maturity stage influenced physical performance in a large sample of young tennis players, with the results showing that Post-PHV players outperformed their Pre- and Circa-PHV peers in jumping ability, linear sprints, and COD ability tests. Moreover, Pre- and Circa-PHV players presented lower CODD% than Post-PHV players in the pro-agility test. From a practical perspective, it seems that the pro-agility test is a highly reliable test to measure the COD ability, including measures of CODD during static and rolling situations, which can be helpful in order to determine how much time it “costs” an athlete to change direction at high entry speeds. Moreover, coaches should be aware of the differences found in the physical performance and consider the practical implications that maturation can have in the long-term development of young tennis players. In this regard, since strength training leads to increases in lean body mass, which may also help improve the ‘athleticism’ of players, the combination of strength training (e.g. resisted sprints, horizontally directed power exercises, and eccentric strength training) and COD workouts (i.e., combining cognitive, physical, and technical aspects with tennis-specific movements in different angles of direction) may be beneficial to improve these qualities, especially to overcome the CODD, which appears to be consistent during all the PHV stages analysed here, and exacerbated in the Post-PHV group. Additional studies are warranted to determine the best training approaches and content (specific to each maturity stage) to meaningfully improve neuromuscular performance in young tennis players, including the CODD.

## References

[1] Reilly T, Morris T, Whyte G. The specificity of training prescription and physiological assessment: A review. J Sports Sci. 2009; 27(6):575–89. 19340630 10.1080/02640410902729741

[2] Murphy AP, Duffield R, Kellett A, Reid M. A comparison of the perceptual and technical demands of tennis training, simulated match play, and competitive tournaments. Int J Sports Physiol Perform. 2016;11(1):40–7. 25849156 10.1123/ijspp.2014-0464

[3] Kovacs MS. Applied physiology of tennis performance. Br J Sports Med. 2006; 40(5):381–5. 16632565 10.1136/bjsm.2005.023309 PMC2653871

[4] Kovalchik SA, Reid M. Comparing matchplay characteristics and physical demands of junior and professional tennis athletes in the era of big data. J Sport Sci Med. 2017; 16(4):489–497. PMC5721178 29238248

[5] Fernandez-Fernandez J, García-Tormo V, Santos-Rosa FJ, Teixeira AS, Nakamura FY, Granacher U, et al. The Effect of a Neuromuscular vs. Dynamic Warm-up on Physical Performance in Young Tennis Players. J Strength Cond Res. 2020;34(10):2776–2784. 32986392 10.1519/JSC.0000000000003703

[6] Ferrauti A, Weber K, Wright PR. Endurance: basic, semi-specific and specific. Strength Cond Tennis London ITF 93–111, 2003.

[7] Madruga-Parera M, Bishop C, Fort-Vanmeerhaeghe A, Beltran-Valls M, Skok O, Romero-Rodríguez D. Interlimb Asymmetries in Youth Tennis Players: Relationships with Performance. J Strength Cond Res. 2019; 34(10):2815–2823. 10.1519/JSC.0000000000003152 31009431

[8] Giles B, Peeling P, Dawson B, Reid M. How do professional tennis players move? The perceptions of coaches and strength and conditioning experts. J Sports Sci. 2019; 37:726–734. 30319029 10.1080/02640414.2018.1523034

[9] Kovacs MS. Movement for tennis: The importance of lateral training. Strength Cond J. 2009; 31(4):77–85.

[10] Fernandez-Fernandez J, Loturco I, Pereira LA, Del Coso J, Areces F, Gallo-Salazar C, et al. Change of Direction Performance in Young Tennis Players: A Comparative Study Between Sexes and Age Categories. J Strength Cond Res. 2022;36(5):1426–1430. 31923020 10.1519/JSC.0000000000003484

[11] Deutsch E, Deutsch SL, Douglas PS. Exercise training for competitive tennis. Clin Sports Med. 1988; 7:417–427. 3292069

[12] Giles B, Peeling P, Reid M. Quantifying Change of Direction Movement Demands in Professional Tennis Matchplay. An Analysis from the Australian Open Grand Slam. J Strength Cond Res. Oct 12. doi: 10.1519/JSC.0000000000003937., 2021. Online Ahead of print. 38320234

[13] Giles B, Peeling P, Kovalchik S, Reid M. Differentiating movement styles in professional tennis: A machine learning and hierarchical clustering approach. Eur J Sport Sci. 2021 Dec 30:1–10. doi: 10.1080/17461391.2021.2006800. Epub ahead of print 34781856

[14] O’Donoghue P and Ingram B. A notational analysis of elite tennis strategy. J Sports Sci. 2001; 19:107–115. 11217009 10.1080/026404101300036299

[15] Martin C, Sorel A, Touzard P, Bideau B, Gaborit R, DeGroot H, et al. Can the Open Stance Forehand Increase the Risk of Hip Injuries in Tennis Players? Orthop J Sport Med. 2020; 8(12):2325967120966297. 10.1177/2325967120966297 PMC7734511 33354579

[16] Cooke K, Quinn A, Sibte N. Testing speed and agility in elite tennis players. Strength Cond J. 2011; 33:69–72.

[17] Leone M, Comtois AS, Tremblay F, Léger L. Specificity of running speed and agility in competitive junior tennis players. Med Sci Tennis. 2006; 11:10–11.

[18] Vuong JL, Fett J, Ulbricht A, Ferrauti A. Physical determinants, intercorrelations, and relevance of movement speed components in elite junior tennis players. Eur J Sport Sci. 2022 Jan 6:1–11. doi: 10.1080/17461391.2021.2005150. Epub ahead of print. 34753414

[19] Ulbricht A, Fernandez-Fernandez J, Mendez-Villanueva A, Ferrauti A. Impact of Fitness Characteristics on Tennis Performance in Elite Junior Tennis Players. J Strength Cond Res. 2016; 30(4):989–998. 26605803 10.1519/JSC.0000000000001267

[20] Nimphius S, Callaghan SJ, Bezodis NE, Lockie RG. Change of Direction and Agility Tests: Challenging Our Current Measures of Performance. Strength Cond J. 2018;40(1):26–38.

[21] Jones PA, Nimphius S. Change of direction and agility. In: Comfort P, Jones PA, McMahon JJ. Performance Assessment in Strength and Conditioning. Routledge, London, UK. 2018. pp. 166–211.

[22] Barber OR, Thomas C, Jones PA, Mcmahon JJ, Comfort P. Reliability of the 505 change-of-direction test in netball players. Int J Sports Physiol Perform. 2016; 11(3):377–80. 26309330 10.1123/ijspp.2015-0215

[23] Nimphius S, Callaghan SJ, Spiteri T, Lockie RG. Change of Direction Deficit: A More Isolated Measure of Change of Direction Performance Than Total 505 Time. J Strength Cond Res. 2016; 30(11):3024–3032. 26982972 10.1519/JSC.0000000000001421

[24] Freitas TT, Pereira LA, Alcaraz PE, Azevedo PHSM, Bishop C, Loturco I. Percentage-Based Change of Direction Deficit: A New Approach to Standardize Time-and Velocity-Derived Calculations. J Strength Cond Res. 2021 Aug 25. doi: 10.1519/JSC.0000000000004118. Online ahead of print. 34446644

[25] Forster JWD, Uthoff AM, Rumpf MC, Cronin JB. Pro-agility unpacked: Variability, comparability and diagnostic value. Int J Sports Sci Coach. 2022; 17(5):1225–1240.

[26] Eriksson A, Johansson FR, Bäck M. Reliability and criterion-related validity of the 20-yard shuttle test in competitive junior tennis players. Open Access J Sport Med. 2015;14; 6:269–76. 10.2147/OAJSM.S86442 PMC4542558 26316829

[27] Sherar LB, Mirwald RL, Baxter-Jones ADG, Thomis M. Prediction of adult height using maturity-based cumulative height velocity curves. J Pediatr. 2005;147:508–514. 16227038 10.1016/j.jpeds.2005.04.041

[28] Mirwald RL, Baxter-Jones ADG, Bailey DA, Beunen GP. An assessment of maturity from anthropometric measurements. Med Sci Sport Exerc. 2002;34:689–694. 10.1097/00005768-200204000-00020 11932580

[29] Davidson T, Jarvis P, Dos’Santos T, Turner A, Bishop C. Modifying the pro-agility test: is the change of direction deficit affected by a rolling start? Prof Strength Cond. 2019; 52:21–29.

[30] Beekhuizen KS, Davis MD, Kolber MJ, Cheng MSS. Test-retest reliability and minimal detectable change of the hexagon agility test. J Strength Cond Res. 2009;23(7):2167–71. 19855348 10.1519/JSC.0b013e3181b439f0

[31] Koo TK, Li MY. A guideline of selecting and reporting intraclass correlation coefficients for reliability research. J Chiropr Med. 2016;15:155–163. 27330520 10.1016/j.jcm.2016.02.012 PMC4913118

[32] Weir JP. Quantifying test-retest reliability using the intraclass correlation coefficient and the SEM. J Strength Cond Res. 2005;19(1):231–40. 15705040 10.1519/15184.1

[33] Freeman PR, Hedges LV, Olkin I. Statistical methods for meta-analysis. Academic press, 2014.

[34] Malina RM, Rogol AD, Cumming SP, Coelho E Silva MJ, Figueiredo AJ. Biological maturation of youth athletes: Assessment and implications. Br J Sports Med. 2015;49(13):852–9. 26084525 10.1136/bjsports-2015-094623

[35] Fernandez-Fernandez J, Canós-Portalés J, Martinez-Gallego R, Corbi F, Baiget E. Effects of Maturation on Lower-Body Neuromuscular Performance in Youth Tennis Players. J Strength Cond Res. 2021; Dec 23 doi: 10.1519/JSC.0000000000004187. Online ahead of print. 36515602

[36] Lloyd RS, Oliver JL. The youth physical development model: A new approach to long-term athletic development. Strength Cond J. 2012;34(3):61–72.

[37] Lloyd RS, Read P, Oliver JL, Meyers RW, Nimphius S, Jeffreys I. Considerations for the development of agility during childhood and adolescence. Strength Cond J. 2013;35(3):2–11.

[38] Morris R, Emmonds S, Jones B, Myers TD, Clarke ND, Lake J, et al. Seasonal changes in physical qualities of elite youth soccer players according to maturity status: comparisons with aged matched controls. Sci Med Footb. 2018;2:272–280.

[39] Beunen G, Malina RM. Growth and biologic maturation: relevance to athletic performance. In: Hebestreit H, Bar-Or O, The Young Athlete. Massachusetts: Blackwell Publ, 2008. pp 3–18

[40] Ford KR, Myer GD, Hewett TE. Longitudinal effects of maturation on lower extremity joint stiffness in adolescent athletes. Am J Sports Med. 2010;38(9):1829–37. 20522830 10.1177/0363546510367425 PMC3968426

[41] Fernandez-Fernandez J, Ulbricht A, Ferrauti A. Fitness testing of tennis players: How valuable is it. Br J Sports Med. 2014;48,(48 Suppl 1):i22–31. 24668375 10.1136/bjsports-2013-093152 PMC3995228

[42] Stewart PF, Turner AN, Miller SC. Reliability, factorial validity, and interrelationships of five commonly used change of direction speed tests. Scand J Med Sci Sports. 2014;24:500–506. 23176602 10.1111/sms.12019

[43] Mann JB, Ivey PA, Mayhew JL, Schumacher RM, Brechue WF. Relationship between agility tests and short sprints: Reliability and smallest worthwhile difference in National Collegiate Athletic Association Division-I football players. J Strength Cond Res. 2016;30:893–900. 26808859 10.1519/JSC.0000000000001329

[44] Fett J, Ulbricht A, Ferrauti A. Impact of Physical Performance and Anthropometric Characteristics on Serve Velocity in Elite Junior Tennis Players. J Strength Cond Res. 2018; 34(1):192–202. 10.1519/JSC.0000000000002641 29912079

[45] Loturco I, Nimphius S, Kobal R, Bottino A, Zanetti V, Pereira LA, et al. Change-of direction deficit in elite young soccer players. Ger J Exerc Sport Res. 2018;48:228–234.

[46] Giles B, Reid M. Applying the brakes in tennis: How entry speed affects the movement and hitting kinematics of professional tennis players. J Sports Sci. 2021;39:259–266. 32935627 10.1080/02640414.2020.1816287

[47] Dos’Santos T, Thomas C, Comfort P, Jones PA. The Effect of Angle and Velocity on Change of Direction Biomechanics: An Angle-Velocity Trade-Off. Sports Med. 2018;48(10):2235–2253. 30094799 10.1007/s40279-018-0968-3 PMC6132493

[48] Harper DJ, Jordan AR, Kiely J. Relationships between eccentric and concentric knee strength capacities and maximal linear deceleration ability in male academy soccer players. J Strength Cond Res. 2021;35:465–472. 29995690 10.1519/JSC.0000000000002739

[49] Fu MC, Ellenbecker TS, Renstrom PA, Windler GS, Dines DM. Epidemiology of injuries in tennis players. Curr Rev Musculoskelet Med. 2018;11(1):1–5. 29340975 10.1007/s12178-018-9452-9 PMC5825333

